# Supplementary material for: High-risk human papillomavirus prevalence in self-collected cervicovaginal specimens from human immunodeficiency virus (HIV)-negative women and women living with HIV living in Botswana
Source: PLoS One. 2020 Feb 13;15(2):e0229086. doi: 10.1371/journal.pone.0229086 (PMC7018080; doi:10.1371/journal.pone.0229086)
Supplement: Attachment — Submitted filename: Response to Reviewers.docx [file pone.0229086.s001.docx]

Response to Reviewers

24 January 2020

PONE-D-19-35746
High-risk human papillomavirus prevalence in self-collected cervicovaginal specimens from human immunodeficiency virus (HIV)-negative women and women living with HIV living in Botswana
PLOS ONE

Please see my responses below in blue.

Best regards,

John E. Varallo

Journal Requirements:

1. When submitting your revision, we need you to address these additional requirements.
Please ensure that your manuscript meets PLOS ONE's style requirements, including those for file naming. The PLOS ONE style templates can be found at
<http://www.journals.plos.org/plosone/s/file?id=wjVg/PLOSOne_formatting_sample_main_body.pdf> and <http://www.journals.plos.org/plosone/s/file?id=ba62/PLOSOne_formatting_sample_title_authors_affiliations.pdf>

2. Please provide additional details regarding participant consent. In the ethics statement in the Methods and online submission information, please ensure that you have specified (1) whether consent was informed and (2) what type you obtained (for instance, written or verbal, and if verbal, how it was documented and witnessed). If your study included minors, state whether you obtained consent from parents or guardians. If the need for consent was waived by the ethics committee, please include this information.

Further details of the informed consent process have been added to the manuscript. Minors were not included in this study.

3. Thank you for including your ethics statement:  "Johns Hopkins School of Public Health (JHSPH) Institutional Review Board IRB00007974".

Please amend your current ethics statement to confirm that your named institutional review board or ethics committee specifically approved this study.

This has been completed.

Once you have amended this/these statement(s) in the Methods section of the manuscript, please add the same text to the “Ethics Statement” field of the submission form (via “Edit Submission”).

This has been completed.

4.  Thank you for stating the following in the Competing Interests section:

Philip E. Castle has received discounted or free HPV tests and assays for research purposes from Roche, Bectin Dickinson, Cepheid, and Arbor Vita Corporation. Otherwise, we have no conflicts of interest to disclose.

Please confirm that this does not alter your adherence to all PLOS ONE policies on sharing data and materials, by including the following statement: "This does not alter our adherence to PLOS ONE policies on sharing data and materials.” (as detailed online in our guide for authors <http://journals.plos.org/plosone/s/competing-interests>). If there are restrictions on sharing of data and/or materials, please state these. Please note that we cannot proceed with consideration of your article until this information has been declared.

This does not alter our adherence to PLOS ONE policies on sharing data and materials.

Please include your updated Competing Interests statement in your cover letter; we will change the online submission form on your behalf.

Please know it is PLOS ONE policy for corresponding authors to declare, on behalf of all authors, all potential competing interests for the purposes of transparency. PLOS defines a competing interest as anything that interferes with, or could reasonably be perceived as interfering with, the full and objective presentation, peer review, editorial decision-making, or publication of research or non-research articles submitted to one of the journals. Competing interests can be financial or non-financial, professional, or personal. Competing interests can arise in relationship to an organization or another person. Please follow this link to our website for more details on competing interests: <http://journals.plos.org/plosone/s/competing-interests>

5. We note that you have included the phrase “data not shown” in your manuscript. Unfortunately, this does not meet our data sharing requirements. PLOS does not permit references to inaccessible data. We require that authors provide all relevant data within the paper, Supporting Information files, or in an acceptable, public repository. Please add a citation to support this phrase or upload the data that corresponds with these findings to a stable repository (such as Figshare or Dryad) and provide and URLs, DOIs, or accession numbers that may be used to access these data. Or, if the data are not a core part of the research being presented in your study, we ask that you remove the phrase that refers to these data.

This has been corrected. Proper tables were added to show this data.

[Note: HTML markup is below. Please do not edit.]

Reviewers' comments:

Reviewer's Responses to Questions

**Comments to the Author**

1. Is the manuscript technically sound, and do the data support the conclusions?

The manuscript must describe a technically sound piece of scientific research with data that supports the conclusions. Experiments must have been conducted rigorously, with appropriate controls, replication, and sample sizes. The conclusions must be drawn appropriately based on the data presented.

Reviewer #1: Yes

Reviewer #2: Yes

2. Has the statistical analysis been performed appropriately and rigorously?

Reviewer #1: Yes

Reviewer #2: Yes

3. Have the authors made all data underlying the findings in their manuscript fully available?

The [PLOS Data policy](http://www.plosone.org/static/policies.action#sharing) requires authors to make all data underlying the findings described in their manuscript fully available without restriction, with rare exception (please refer to the Data Availability Statement in the manuscript PDF file). The data should be provided as part of the manuscript or its supporting information, or deposited to a public repository. For example, in addition to summary statistics, the data points behind means, medians and variance measures should be available. If there are restrictions on publicly sharing data—e.g. participant privacy or use of data from a third party—those must be specified.

Reviewer #1: Yes

Reviewer #2: Yes

4. Is the manuscript presented in an intelligible fashion and written in standard English?

PLOS ONE does not copyedit accepted manuscripts, so the language in submitted articles must be clear, correct, and unambiguous. Any typographical or grammatical errors should be corrected at revision, so please note any specific errors here.

Reviewer #1: Yes

Reviewer #2: Yes

5. Review Comments to the Author

Please use the space provided to explain your answers to the questions above. You may also include additional comments for the author, including concerns about dual publication, research ethics, or publication ethics. (Please upload your review as an attachment if it exceeds 20,000 characters)

Thank you very much for the insightful and useful feedback to help make this a stronger manuscript. Please see below my responses.

Reviewer #1: This research article on hrHPV prevalence among HIV negative and WLWH in Botswana based on self-collected sample is a pilot demonstration study that documents success of self-collection in the Botswana context and provides new and insightful data on association of hrHPV and HIV. This is an impactful study and significant to the field of HIV/HPV and advances science on the use of HPV based screening in limited resource setting both among HIV negative and WLWH.


1. Methods (population)- Recruitment at various facility based on site (health facility vs. community) is difficult to follow based on text presented. It is unclear if two of the recruitment sites only had community health based outreach recruitment?

Thank you for pointing that out. I hope the following is clearer.

All five health facilities conducted recruitment at their respective facilities, while two health facilities (B and E) also conducted recruitment in the community during health outreach activities. In these two facilities, recruitment was planned to be half and half from the health facility and in the community each serves through existing integrated community health outreaches, but 70% were recruited by community outreach for one facility (B).

2. Result (Table 4)- Data as presented in the text does not match table 4. Furthermore, the numbers as presented as total visible lesion do not add up. Please clarify

Thank you very much for catching that. The text was correct. It has been reviewed and corrected in the table, which is now Table 5, since we added tables to capture ‘data not shown’.

Reviewer #2: The manuscript addresses a current topic as self collection is an important way to increase screening coverage if HPV testing is available in a low resource setting . Overall the manuscript was well written and worthy of publication. Some minor comments noted:
Introduction should reference that self collections was previously found to be acceptable to women in Botswana (ex reference previous studies on acceptability). .

This is an excellent point regarding acceptability of self-collection, in other settings. At the time of our study, however, data on acceptability in Botswana was not available. However, our study did collect that information and we are in the process of writing a separate paper on the feasibility and acceptability aspects of the study.

Page 9 Methods: last paragraph beginning with "The outcome of VAT.......HPV16+ ELSE, HPV negative........".
Does ELSE imply versus: please clarify

Agree – versus is more clear and this has been changed.

Page 12 Results section: paragraph following Table 2 that begins with "Interestingly, the hrHPV prevalence differed between......"
This was difficult to follow since # clinic vs # community referrals were not identified for any of the clinics (A,B, C, D, or E). Therefore it was difficult to assess the prevalence data given, without knowing n values.

 This was addressed through the above comment, and copied again below.

All five health facilities conducted recruitment at their respective facilities, while two health facilities (B and E) also conducted recruitment in the community during health outreach activities. In these two facilities, recruitment was planned to be half and half from the health facility and in the community each serves through existing integrated community health outreaches, but 70% were recruited by community outreach for one facility (B).

6. PLOS authors have the option to publish the peer review history of their article ([what does this mean?](https://journals.plos.org/plosone/s/editorial-and-peer-review-process#loc-peer-review-history)). If published, this will include your full peer review and any attached files.


If you choose “no”, your identity will remain anonymous but your review may still be made public.


**Do you want your identity to be public for this peer review?** For information about this choice, including consent withdrawal, please see our [Privacy Policy](https://www.plos.org/privacy-policy).

Reviewer #1: No

Reviewer #2: No


[NOTE: If reviewer comments were submitted as an attachment file, they will be attached to this email and accessible via the submission site. Please log into your account, locate the manuscript record, and check for the action link "View Attachments". If this link does not appear, there are no attachment files to be viewed.]


While revising your submission, please upload your figure files to the Preflight Analysis and Conversion Engine (PACE) digital diagnostic tool, <https://pacev2.apexcovantage.com/>. PACE helps ensure that figures meet PLOS requirements. To use PACE, you must first register as a user. Registration is free. Then, login and navigate to the UPLOAD tab, where you will find detailed instructions on how to use the tool. If you encounter any issues or have any questions when using PACE, please email us at [figures@plos.org](mailto:figures@plos.org). Please note that Supporting Information files do not need this step.
